# La place du traitement non opératoire des contusions abdominales dans les pays en voie de développement

**DOI:** 10.11604/pamj.2015.20.132.1212

**Published:** 2015-02-16

**Authors:** Khalid Rabbani, Youssef Narjis, Abdelouahed Louzi, Redouane Benelkhaiat, Benacer Finech

**Affiliations:** 1 Département de chirurgie générale, CHU Mohamed VI, Université Caddi ayyad, Marrakech, Maroc

**Keywords:** Contusion abdominale, traitement non opératoire, hemopéritoine, pneumopéritoine, polytraumatisée, transfusion, abdominal contusion, non-operative treatment, haemoperitoneum, pneumoperitoneum, polytrauma, transfusion

## Abstract

Le traitement non opératoire des contusions de l'abdomen représente une approche thérapeutique nouvelle des traumatismes fermés de l'abdomen. La disponibilité d'un plateau technique performant constitue classiquement une des principales conditions pour la réussite de cette attitude. Nous essayons d’étudier les différents aspects épidémiologiques, diagnostiques et thérapeutiques de cette affection dans le contexte d'un pays en voie de développement, à travers les résultats d'une série de 106 malades. une série de 106 patients est prise en charge par le traitement non opératoire. La gravité des lésions est appréciée par les données cliniques et paracliniques, ainsi que la morbidité et la mortalité sont analysées. les accidents de la circulation étaient la première cause des contusions abdominales, dans notre contexte. Les lésions hépatiques étaient les plus fréquentes (65% des cas), suivit des lésions spléniques chez 19 patients. La notion de polytraumatisme était très fréquente. La durée moyenne de surveillance était de 9,5 jours. Seize malades étaient transfusés, et le nombre moyen de culots globulaires (CG) était de 3,5 culots par patient. Une laparotomie en urgence était indiquée chez 10 patients (9.4%) devant l'instabilité hémodynamique. Trois malades ont nécessité un traitement chirurgical secondaire. On avait noté un taux de mortalité de 3.7% soit 4 cas. il semble à partir de notre expérience que l'abstention chirurgicale peut constituer, dans des conditions strictes de surveillance, une alternative thérapeutique de référence dans les pays en voie de développement, sûre et justifiée à une chirurgie d'urgence toujours difficile.

## Introduction

Depuis quelques années, le traitement non opératoire a connu un développement important dans la gestion des traumatismes de l'abdomen, car il permet une meilleure survie et un meilleur taux de sauvetage d'organe. Il évite de surcroît aux patients les complications d'une laparotomie inutile. Le chirurgien a toujours sa place auprès des patients, mais le scalpel semble baisser pavillon devant le traitement non chirurgical [1].

Le but de ce travail ne cherche pas à valider la méthode thérapeutique elle-même, mais se propose d’évaluer la faisabilité du traitement conservateur, lors des contusions abdominales chez l'adulte dans le contexte d'un pays en voie de développement.

## Méthodes

Ce travail a porté sur 106 cas de contusions abdominales, hospitalisés au service de chirurgie générale sur une période de six ans allant du 1er janvier 2004 à la fin décembre 2009. Les dossiers des patients ont été analysés selon une fiche d'exploitation préétablie.

## Résultats

**Figure 1:**
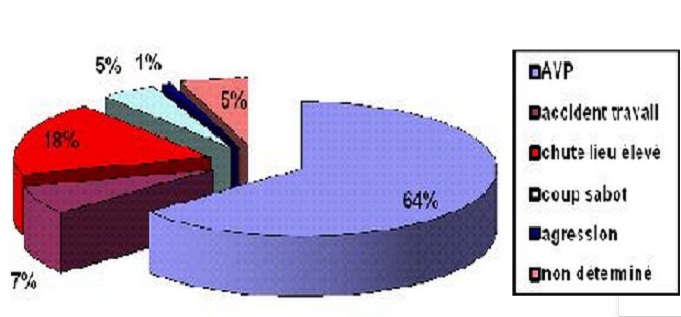
Étiologies des contusions abdominals

**Figure 2:**
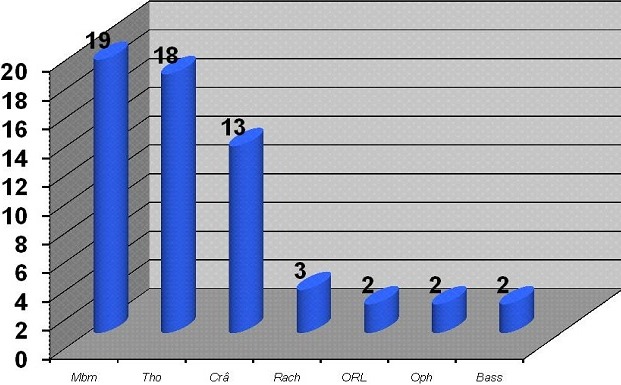
Lésions associées à la contusion abdominale

La série globale comportait 106 cas. L’âge moyen était de 30,8 ans (extrêmes 10 à 73 ans). Il y avait 91 hommes (85.8%) et 15 femmes (14.1%). Les circonstances du traumatisme (Figure 1), étais dominés par les accidents de la voie publique (64%) suivis des chutes d'un lieu élevé (18%), des accidents de travail (7%), des coups de sabot (5%) et des agressions (1 cas). Le mécanisme était direct chez 88% des patients. Treize patients étaient en état de choc et 3 patients en détresse respiratoire. Les signes fonctionnels étaient dominés par la douleur qui était abdominale dans 87 cas et basithoracique dans 8 cas. Les autres signes fonctionnels étaient des vomissements chez 14 patients (13%), une distension abdominale chez 5 patients (5%) et une hémorragie digestive (2 cas). L'examen avait montré une défense abdominale chez 36% des malades (38 cas) et une sensibilité abdominale chez 71.7% d'entre eux (Tableau 1). Un polytraumatisme était retrouvé chez 25.5% des patients (Figure 2), avec des traumatismes des membres (19 cas), thoracique (18 cas) et crânien (13 cas). Le bilan biologique avait trouvé une anémie normochrome dans 40 cas, une cytolyse dans 26 cas et une cholestase dans 4 cas. La radiographie d'abdomen sans préparation avait objectivé des niveaux hydroaériques dans 4 cas et un pneumopéritoine dans 2 cas, alors que la radiographie du thorax avait montré un épanchement pleural dans 10 cas, des fractures de côtes dans 6 cas, des foyers de contusion pulmonaire dans 3 cas et un pneumothorax dans 3 cas. L’échographie abdominale était réalisée en urgence chez tous les patients. Elle avait montré un épanchement intrapéritonéal dans 83 cas (de moyenne abondance dans 51 cas) et des lésions viscérales dans 60 cas. Il s'agissait de lésions hépatiques dans 27 cas (16 contusions, 6 fractures et 5 hématomes) et des lésions spléniques dans 21 cas (14 contusions, 5 fractures et 2 hématomes) (Tableau 2). Un scanner abdominal était réalisé dans 57 cas (53.7%). Il avait montré un épanchement intra péritonéal dans 42 cas, un épanchement pleural dans 9 cas et un pneumopéritoine dans 3 cas. Les lésions hépatiques étaient les plus fréquentes (65% des cas), à type de contusion hépatique dans 24 cas, fracture hépatique dans 11 cas et hématome sous capsulaire dans 2 cas. Les lésions spléniques étaient retrouvées chez 19 patients, il s'agissait de contusion dans 6 cas et de fracture dans 13 cas. D'autres lésions étaient retrouvées, il s'agissait de 2 cas de lésions pancréatiques, 7 cas de lésion rénale, 3 plaies du grêle, un hématome de la paroi vésicale, un hématome de la paroi colique et un cas de rupture diaphragmatique.

**Tableau 1:** Les différents éléments de l'examen abdominal

Signes physiques	Nombre de cas	Pourcentage%
Distension abdominale	01	0.9
Ecchymoses, Hematomes	12	11.3
Sensibilité abdominale localisée	46	43.3
Sensibilité abdominale généralisée	30	28.3
Défense abdominale localisée	23	21.7
Décense abdominale généralisée	15	14
Contracture abdominale	02	1.8

**Tableau 2:** Les différentes lésions viscérales au cours des contusions abdominales à l’échographie abdominale

Lésion viscérale		Nombre de cas	Pourcentage%
**Contusions hépatiques**	Segment IV	6	10
	Segment V	11	18.3
	Segment VI	6	13.3
	Segment VII	7	11.6
	Segment VIII	14	23.3
**Lésions de rate**	Contusion	14	23.3
	Fracture	5	8.3
	Hématome	2	3.3

La prise en charge de ces patients était primaire pour 80 d'entre eux (75.4%) et par transfert pour 26 cas (24.6%). Une transfusion sanguine de culots globulaires était nécessaire chez 16 de nos patients (15%), et le nombre moyen de culots globulaires (CG) était de 3,5 culots par patient. Une laparotomie en urgence était indiquée chez 10 patients (9.4%) devant l'instabilité hémodynamique malgré les mesures de réanimation dans 7 cas, et devant des signes de péritonite dans 3 cas. Un traitement non opératoire était préconisé chez 96 patients (90.6%). Il était adopté devant la stabilité hémodynamique et aussi devant l'absence de pneumopéritoine, de contracture abdominale et de lésions cérébrales. Une laparotomie secondaire était nécessaire chez seulement 3 patients (3%) devant l'apparition de signes de péritonite dans un cas et l'altération secondaire de l’état hémodynamique dans 2 cas. On avait noté un taux de mortalité de 3.7% soit 4 cas. Des complications étaient survenues chez 7 patients avec 2 cas de bilome, 3 cas d'hémobilie et 2 cas de surinfection de la plaie chirurgicale. L’évolution était jugée favorable chez 99 patients avec une durée moyenne d'hospitalisation de 9.5 jours.

## Discussion

Le Traumatisme abdominal Fermé est un défi diagnostique et thérapeutique pour le médecin. Par opposition au traumatisme abdominal pénétrant, le traumatisme fermé présente un taux de mortalité induit plus important du fait qu'il est difficile à diagnostiquer et qu'il est souvent associé à d'autres traumatismes intra- ou extrapéritonéaux [2, 3].

Les données épidémiologiques générales de notre population semblent être concordantes avec les données de la littérature. Celle-ci montre que les contusions abdominales touchent en premier le sujet jeune, de sexe masculin [4, 5]. Les AVP constituent la première étiologie, suivis des chutes d'un lieu élevé [6–8]. La douleur constitue un signe d'appel primordial dans les contusions abdominales, elle est souvent localisée suivant la projection de l'organe lésé sur la paroi abdominale. Les vomissements témoignent d'une irritation péritonéale. La distension abdominale, l'hémorragie digestive ainsi que l'hématurie sont d'autres signes fonctionnels à rechercher de façon systématique. L'examen clinique, malgré ses insuffisances et ses difficultés de réalisation, est jugé fondamental par la plupart des auteurs permettant d'apprécier: la fonction ventilatoire, température, recherche de douleurs, météorisme, trouble du transit, signes d'anémie, mesure du pouls, de la tension, de la diurèse [9]. Sur le plan biologique, les principaux examens à prescrire sont: l'hématocrite, l'hémoglobine, la numération formule sanguine, un bilan hépatique, le dosage des lipases ou des amylases. L’échographie constitue un outil essentiel permettant de faire le diagnostic des collections liquidiennes intra et rétro péritonéales et celui des lésions d'organes pleins ainsi que leur surveillance en cas de traitement conservateur [9]. Le lavage péritonéal peut être une autre option possible en l'absence d’échographie ou si l’état d'un patient est instable, mais il est de moins en moins pratiquer [10]. Quant à la tomodensitométrie abdominale avec contraste intraveineux, elle est devenue l'examen de référence pour le diagnostic et le suivi des patients présentant des traumatismes abdominaux importants. L'angiographie demeure un élément d’évaluation en second plan, parfois essentielle pour confirmer une hémorragie active ou retardée et pour procéder à l'embolisation, au besoin [11].

Au terme de ces examens, l'identification du bilan lésionnel permet de distinguer des lésions élémentaires et des lésions spécifiques. La chirurgie en urgence reste la règle chez le traumatisé abdominal dont l'hémodynamique est instable malgré une réanimation bien menée, ou en cas de lésion évidente d'organe creux. La laparotomie par voie médiane sera dans ce cas préférable. La mini laparotomie représente l'alternative entre l'exploration par une laparotomie classique et la laparoscopie. Elle n'est plus de mise si cette dernière peut être réalisée. La laparotomie écourtée correspond à la réalisation d'un geste le plus rapide possible et donc incomplet, limité au constat des lésions et au contrôle sommaire d'une hémorragie active et/ou d'une fuite digestive, suivis de la fermeture de la laparotomie pour laisser la place au plus vite à la réanimation qui s'impose [12]. Parallèlement, certaines études récentes révèlent le succès du traitement conservateur des contusions abdominales chez l'adulte. Il est réalisable dans 55% à 80% des cas avec un taux de réussite de l'ordre de 60% ou 70% [13], dans l'optique de diminuer les interventions chirurgicales et ainsi d'augmenter les chances de préserver les organes pleins, avec possibilité d'intervenir secondairement, pour permettre une prise en charge moins effractive d'un traumatisme abdominal. Cette option nécessite une surveillance chirurgicale et médicale clinique vigilante du traumatisé de l'abdomen initialement non opéré, quelque soit l'organe atteint [14]. Cette surveillance « armée » comporte la disponibilité d'une équipe de chirurgiens, d'anesthésistes et de radiologues, disposant d'outils diagnostiques performants. L'approche non opératoire est loin d’être non chirurgicale: à tout moment, le blessé peut présenter des signes évoquant la poursuite d'une hémorragie, une atteinte d'organe creux ou du pancréas, un syndrome du compartiment abdominal [15, 16]. Ses indications augmentent grâce à l'apport de l'embolisation par voie artérielle. Cette technique a été initialement développée pour les patients atteints d'un traumatisme retropéritonéal grave, essentiellement rein et bassin, et elle est maintenant réalisable chez des patients présentant des traumatismes hépatiques et, plus récemment, spléniques. Les échecs du traitement par observation surviennent principalement au cours des premières 24 heures [17, 18]. Cette donnée pourrait être à prendre en compte pour l'orientation de l'observation des patients.

## Conclusion

Le paysage du traumatisme abdominal évolue. Nous serons dorénavant plus conservateurs en présence d'un traumatisme fermé de l'abdomen y compris dans le contexte d'un pays en voie de développement, à condition de disposer d’ une équipe multidisciplinaire associant des équipes médicales, chirurgicales et radiologiques disposant d'un minimum de moyens permettant la surveillance qui préviendra probablement beaucoup de laparotomies d'urgence.
